# Adjuvanting influenza hemagglutinin vaccine with a human pulmonary surfactant-mimicking synthetic compound SF-10 induces local and systemic cell-mediated immunity in mice

**DOI:** 10.1371/journal.pone.0191133

**Published:** 2018-01-25

**Authors:** Hyejin Kim, Takashi Kimoto, Satoko Sakai, Etsuhisa Takahashi, Hiroshi Kido

**Affiliations:** Division of Enzyme Chemistry, Institute for Enzyme Research, Tokushima University, Tokushima, Japan; University of South Dakota, UNITED STATES

## Abstract

We reported previously that intranasal instillation of a synthetic human pulmonary surfactant with a carboxy vinyl polymer as a viscosity improver, named SF-10, shows potent adjuvanticity for humoral immunity in mice and cynomolgus monkeys. SF-10 effectively induces influenza hemagglutinin vaccine (HAv)-specific IgA in nasal and lung washes and IgG in sera with their neutralizing activities. Since CD8^+^ T cell-mediated protection is an important requirement for adaptive immunity, we investigated in this study the effects of SF-10 with antigen on local and systemic cell-mediated immunity. Nasal instillation of ovalbumin, a model antigen, combined with SF-10 efficiently delivered antigen to mucosal dendritic and epithelial cells and promoted cross-presentation in antigen presenting cells, yielding a high percentage of ovalbumin-specific cytotoxic T lymphocytes in the nasal mucosa, compared with ovalbumin alone. Nasal immunization of HAv-SF-10 also induced HAv-specific cytotoxic T lymphocytes and upregulated granzyme B expression in splenic CD8^+^ T cells with their high cytotoxicity against target cells pulsed with HA peptide. Furthermore, nasal vaccination of HAv-SF-10 significantly induced higher cytotoxic T lymphocytes-mediated cytotoxicity in the lungs and cervical lymph nodes in the early phase of influenza virus infection compared with HAv alone. Protective immunity induced by HAv-SF-10 against lethal influenza virus infection was partially and predominantly suppressed after depletion of CD8^+^ and CD4^+^ T cells (induced by intraperitoneal injection of the corresponding antibodies), respectively, suggesting that CD4^+^ T cells predominantly and CD8^+^ T cells partially contribute to the protective immunity in the advanced stage of influenza virus infection. These results suggest that SF-10 promotes effective antigen delivery to antigen presenting cells, activates CD8^+^ T cells via cross-presentation, and induces cell-mediated immune responses against antigen.

## Introduction

Seasonal influenza A virus (IAV) infection is a major cause of morbidity and mortality, estimated to be responsible for 3–5 million cases of severe illness and ~259,000–500,000 deaths worldwide per annum [1]. The currently available influenza vaccines administered intramuscularly or subcutaneously induce a predominantly IgG-mediated protection in the systemic immune compartment and significantly reduce hospitalization and deaths when they match antigenically the circulating viral strains [2]. However, these vaccinations neither results in adequate induction of antiviral secretory IgA (SIgA), which provides a wide cross-protection, nor efficient prevention of infection at the airway mucosa [2–4], or cell-mediated responses with cross-protection in the early phase of infection in the respiratory tract [4–6].

Since induced antibodies have no access to intracellular viruses, virus antigen-specific cytotoxic T lymphocytes (CTL) play important roles in killing virus-infected cells and thus limiting viral spread and contributing to the eventual clearance of infection and virus expansion [5, 6]. In addition, CTL can recognize and target the internal virus proteins, which are highly conserved, unlike surface proteins [2, 5, 6], and their cross-reactive cellular immunity is efficient and decreases the severity of illness [5]. For the development of efficient influenza vaccine, CTL induction with a heterosubtypic immunity is strongly desired in addition to the humoral immunity.

Mucosal vaccines and adjuvants have been studied for over 40 years [2, 7, 8], but many have been found ineffective or have safety problems [8]. Recently, the cold-adaptive live flu intranasal vaccines, Flumist^®^ and Nasovac^®^, have become available in the USA and Europe. These vaccines induce both humoral and cellular immunity [2], but concern about their safety have already be raised [9, 10], and both have not been approved for use in children under 2 years of age [9].

To overcome the issues of safety and efficacy in mucosal vaccine, we reported previously that bovine pulmonary surfactant, Sufracten^®^, which has been widely used as a natural pulmonary surfactant replacement medicine in premature babies with respiratory distress syndrome, is a useful and safe mucosa adjuvant with potent humoral immune responses [11–13]. However, mucosal vaccines do not often induce sufficient immunity; mainly due to the poor efficiency of antigen (Ag) uptake across the nasal mucosa due to rapid mucociliary clearance. The lung surfactant has remarkable characteristics of infiltration of the airway mucosa and permeation into alveolar cells, macrophages and dendritic cells (DCs), with rapid metabolism in the lungs [14, 15]. We also reported that Surfacten^®^ acts as an efficient Ag delivery vehicle to antigen presenting cells (APCs), when Ag binds to its liposome surface, and the prolongation of Ag duration in nasal cavity by Surfacten^®^ enhances both local and systemic immunity [12], although Surfacten^®^ by itself has no stimulatory effect on DCs [11], unlike many of mucosal adjuvants reported to stimulate DCs.

To prepare a synthetic mucosal adjuvant as a substitution for the natural compound Surfacten^®^, we selected three major lipid constituents and surfactant protein C (SP-C) of the human pulmonary surfactant for mucosal adjuvanticity, and developed a synthetic pulmonary surfactant (SSF) consisting of the major lipids and SP-C related cationic hydrophobic peptide K6L16 [13]. Furthermore, we added 0.5% carboxy vinyl polymer (CVP), as an additive, to the Ag-SSF complex to increase the viscosity and the final solution was renamed Ag-SF-10. Intranasal administration of Ag-SF-10 resulted in significant enhancement of induction of Ag-specific nasal SIgA and serum IgG, compared with Ag alone in mice [13]. In addition, nasal administration of Ag-SF-10 in young cynomolgus monkeys also elicited significantly higher Ag-specific serum IgG and nasal SIgA with cross-neutralizing activities compared with Ag alone, but did not show adverse effects (e.g., body weight loss, fever, nasal discharge, changes in peripheral blood leukocyte and platelet counts, or pathological changes in various organs) [16]. However, studies on the effects of nasal administration of Ag-SF-10 on induction of T cell-mediated protective immunity locally and systemically remain to be performed.

In the present study, we evaluated SF-10 adjuvanticity in the induction of T cell-mediated immune response, a key issue other than humoral immune responses for adaptive immunity in mucosal vaccine. Specifically, the study was designed to determine the effects of nasal administration of SF-10 on Ag delivery to immune cells in the nasal mucosa; activation of CD8^+^ T cell response via cross-presentation, and induction of CTL in the nasal mucosa and spleen; cytotoxicity assay *in vitro/vivo*; roles of CD8^+^ and CD4^+^ T cells in the survival of mice immunized with IAV hemagglutinin vaccine (HAv)-SF-10 after lethal dose of IAV infection.

## Materials and methods

### Mice

Female C57BL/6 and BALB/c mice (age 6–8 weeks) were purchased from Japan SLC (Shizuoka, Japan). Transgenic OT-1 mice were from Jackson Laboratory (Bar Harbor, ME). All animals were maintained under specific-pathogen-free conditions. The mice were treated according to the Guide for the Care and Use of Laboratory Animals (NIH Publication No. 85–23, 1996). For virus challenge experiments, mice were infected with IAV by intranasal instillation and then monitored daily for body weight and visually for signs of clinical diseases, such as body inactivity, ruffled fur, labored respiration and huddling behavior. Mice that lost ≥30% of baseline body weight and/or displayed evidence of pneumonia were euthanized by overdose of intraperitoneal injection of ketamine and xylazine. The study was approved by the Animal Care Committee of Tokushima University (# T27-109).

### Preparation of Ag-SF-10

Split product of influenza HAv of IAV/California/7/2009(H1N1) (0.54 mg HA per 1 mg protein) was provided by Kitasato Daiichi Sankyo Vaccine Co. (Tokyo, Japan). SSF was prepared by mixing three major lipids of pulmonary surfactant, 1,2-dipalmitoyl-phosphatidylcholine, phosphatidylglycerol, and palmitic acid, and a cationic hydrophobic peptide K6L16, at a weight ratio of 75:25:10:2 [13]. SSF was mixed with HAv or ovalbumin (OVA) as Ags, at a SSF phospholipid:Ag protein ratio of 10:1 at 42°C, and the stable Ag-SSF complex was prepared by lyophilization and stored at -20°C until use. Before nasal administration, lyophilized Ag-SSF was dissolved with 0.5% CVP in saline by mild mixing [13]. The final solution was used as Ag-SF-10.

### Immunization and analysis of T cell responses

Mice were anesthetized by intraperitoneal injection of ketamine (62.6 mg/kg) and xylazine (12.4 mg/kg), and immunized on days 0, 14, and 28 by intranasal instillation of 6 μl (3 μl on each nostril) of Ag-SF-10, Ag in saline or saline alone. Another group of mice was also immunized with subcutaneous injection of Ag in 100 μl saline under the same inoculation schedule. As a positive control group, mice were immunized intranasally with 6 μl of 0.1 to 10 μg of Ag combined with 10 μg polyI:C in saline under the same administration schedule.

For detection of Ag-specific CTL in nasal mucosa, nasal tissues of C57BL/6 mice immunized with OVA-SF-10 or BALB/c mice immunized with HAv-SF-10 were isolated, and enzymatically treated by Accutase^®^ cell detachment solution (Innovative Cell Technologies) according to the protocols provided by the manufacturer. Nasal lymphocytes were purified as reported previously [17], and then stained with anti-mouse CD8 antibody (Ab) (MBL Co., Nagoya, Japan) and H-2K^b^ OVA Tetramer (MBL) in mice immunized with OVA-SF-10. To assess CD8^+^ T cell response, spleens from each group of mice intranasally inoculated with HAv with or without SF-10 were isolated and single cell suspensions were prepared [17]. These splenocytes were then incubated with or without 10 μg/ml HAv for 72 h. For detection of granzyme B (GrB) producing CD8^+^ T cells, the splenocytes were further incubated for 6 h in the presence of BD Golgiplug (BD Biosciences, Franklin Lakes, NJ), stained with anti-mouse CD8α Ab (BD Biosciences), fixed and permeabilized by BD Cytofix/Cytoperm^™^ (BD Biosciences), and stained with anti-mouse GrB Ab (BioLegend, San Diego, CA).

Flow cytometry analysis was performed using FACSCalibur or FACSVerse (BD Biosciences) and Flowjo software (Tree Star Inc., Ashland, OR).

### Measurement of Ag uptake into nasal mucosa cells

Alexa647-labeled OVA (Thermo Fisher Scientific, Middletown, VA) with or without SF-10 was administrated intranasally into BALB/c mice as described above. At 15 min after vaccination, the nasal tissues were isolated and cryosection slides were prepared [12, 18]. The slices were then stained with Hoechst33342 (Doujin Chemical Co., Kumamoto, Japan) and fluorescence images were acquired with a microscope (BX-X700; Keyence Co., Osaka, Japan). At 60 min after vaccination, nasal cells were also isolated by Accutase^®^, stained with anti-mouse CD326, CD11c and CD8α Ab (BioLegend), and analyzed by flow cytometry.

### *In vitro/vivo* CD8^+^ T cell activation assays

CD8^+^ T cells were isolated from the spleen and inguinal and cervical lymph nodes (CLNs) of OT-1 mice, which are transgenic mice for a TCR specific for OVA_257-264_ peptide (H-2K^b^), and further purified using CD8^+^ negative selection system (STEMCELL Technologies Inc., Tukwila, WA), and used as OT-1 CD8^+^ T cells. Bone marrow-derived dendritic cells (BMDCs) were prepared as described previously [12]. BMDCs (2 × 10^5^) in 96-well plate were stimulated for 2 h with OVA or OVA-SSF at the indicated concentrations. After washing the cells, the culture medium was replaced with a fresh complete Roswell Park Memorial Institute medium (RPMI) (cRPMI), consisting of RPMI 1640 complemented with 10 mM HEPES, 1 mM sodium pyruvate, 1% MEM non-essential amino acids solution, 14.3 μM 2-mercaptoethanol, 50 μg/ml gentamycin and 10% heat-inactivated fetal bovine serum (FBS). After 24 h-stimulation, the cells were co-incubated with 1 × 10^5^ OT-1 CD8^+^ T cells in cRPMI with 1% FBS, and IL-2 concentration in the culture medium was measured using the mouse IL-2 ELISA kit (eBioscience, San Diego, CA).

For *in vivo* assay, carboxyfluorescein diacetate (CFSE)-labelled OT-1 CD8^+^ T cells (5 × 10^6^) were transferred intravenously into naïve C57BL/6 mice. After 24 h-treatment, OVA-SF-10, OVA or saline was administrated intranasally, and lymphocytes from CLNs of the vaccinated mice were isolated at day 4 after the vaccination. The lymphocytes were stained with anti-CD44 Ab (Biolegend) and analyzed by flow cytometry.

### *In vitro/vivo* cytotoxicity assay

Murine macrophage cell line J774A.1 derived from a tumor in female BALB/c mouse was obtained from the American Type Culture Collection. The cells were cultured in Dulbecco’s modified Eagle medium (DMEM) supplemented with 10% FBS and 50 μg/ml gentamycin. J774A.1 cells (1×10^6^) were pulsed with 1 μM HA peptide 533IYSTVASSL541 at 37°C for 1 h and used as target cells. Splenocytes of BALB/c mice immunized with the indicated amounts of HAv were isolated, re-stimulated with 10 μg/ml HAv and 15 U/ml murine IL-2 for 3 days and used as effector cells. Cytolysis of target cells was analyzed by measurement of the released amount of cytosol lactate dehydrogenase (LDH) in culture media by using CytoTox96 nonradioactive cytotoxicity assay kit (Promega, Madison, WI) according to the instructions provided by the manufacturer. Effector cells (2 × 10^5^) and J774A.1 target cells (1 × 10^4^) pulsed with or without HA peptide, were co-cultured at 37°C for 5 h in V-bottom 96-well plate. After incubation, the culture supernatant was collected for measurement of LDH and the percentage of lysis was calculated according to the protocol supplied by the manufacturer.

For the *in vivo* cytotoxicity assay, BALB/c mice were immunized intranasally with saline, HAv (10 μg) and HAv (10 μg)-SF-10 as described above. Two weeks after the last immunization, all mice were infected with PR/8 at a dose of 100 × LD_50_ (100 PFU). HA peptide-unpulsed CFSE^low^ and pulsed CFSE^high^ splenocytes were prepared using 1 μM HA peptide as described [19], mixed equally, and the cells (5 × 10^6^) were transferred intravenously into the infected mice at 24 h after PR/8 infection. One day after adoptive transfer, lymphocytes from CLNs and lungs were isolated, prepared as single dispersion cells [17], and analyzed for the presence of CFSE-labeled cells by flow cytometry. The percent-specific lysis was determined by loss of the peptide-pulsed CFSE^high^ population compared with the unpulsed CFSE^low^ population using the formula: 100-{(absolute no. peptide-pulsed CFSE^high^ population/absolute no. peptide-unpulsed CFSE^low^ population) × 100)}.

### Protection against PR/8 infection in mice depleted of CD8^+^ and CD4^+^ T cells after immunization with HAv-SF-10

Four weeks after the third intranasal immunization with HAv-SF-10, HAv, or saline (*n* = 5), mice were divided into three groups; the CD4^+^ T cell-depleted group, CD8^+^ T cell-depleted group, and the non-CD4^+^/CD8^+^ T cell-depleted group. Mice of all the three groups were infected with 20 μl of PR/8 at a lethal dose of 20 × LD_50_ into one nostril. For the CD4^+^ and CD8^+^ T cell depleted groups, mice were intraperitoneally injected 4 times with 100 μg of anti-CD8α Ab (53–6.7 clone, BioLegend) and anti-CD4 Ab (GK1.5 clone, BioLegend), respectively, at the indicated time points. The initial injection of these Abs for T cell depletion was at day 4 before infection. Mice of all groups were monitored every day for 2 weeks for survival and loss of body weight. To confirm the CD4^+^ and CD8^+^ T cell depletion by the method employed, mice were treated with these depletion Abs in the same time schedule described above in the separate experiment. At day 8 after the initial injection of these Abs, CD4^+^ and CD8^+^ T cells in the splenocytes were detected with anti-CD4 (53.6.7 clone, BioLegend) and anti-CD8α (53–6.7 clone, BD) Abs by flow cytometry.

### Statistical analysis

Values were expressed as mean ± SEM. Statistical significance of the differences between experimental groups was calculated by ANOVA with a Bonferroni posttest or an unpaired Student’s *t*-test (KaleidaGraph 4.5 software; Synergy Software, Reading, PA). Survival rate was analyzed by the Kaplan-Meier and log-rank tests. *P* values less than 0.05 were considered statistically significant.

## Results

### SF-10 increases Ag-delivery to antigen presenting cells in nasal mucosa

**Fig 1:**
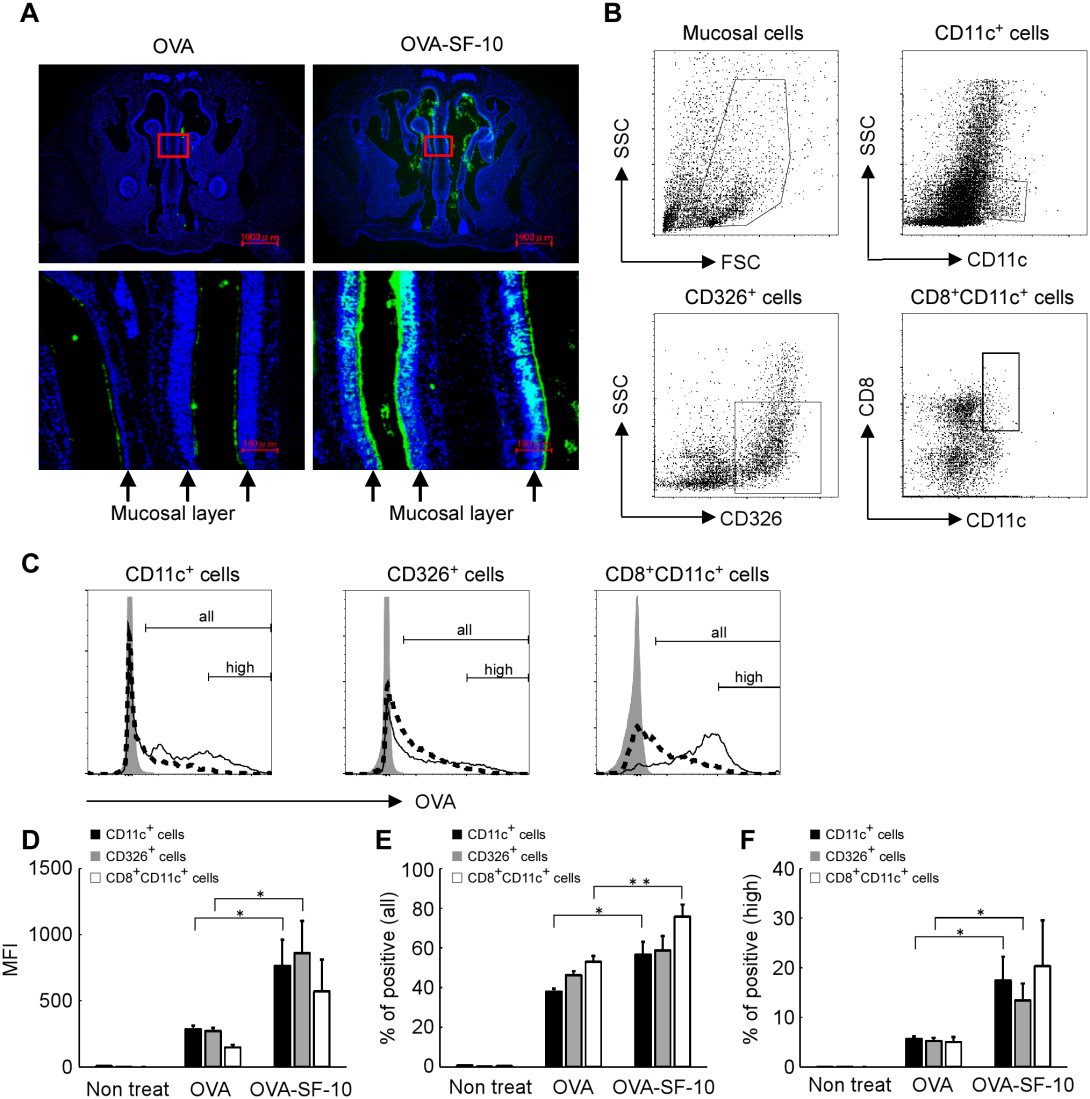
SF-10 enhances Ag delivery to the nasal mucosa. Alexa647-labeled OVA or Alexa647-labeled OVA-SF-10 were instilled intranasally in BALB/c mice. Cryosections of nasal tissues were prepared 15 min after immunization (A). *Top panels*: representative distribution of Alexa647-labeled OVA (green) in the nasal cavity, *bottom panels*: high enlargement of the red square in the top panels. Nuclei stained with Hoechst33342 (blue). Arrow: nasal mucosal layer. After administration of Alexa647-labeled OVA and Alexa647-labeled OVA-SF-10 for 60 min, mucosal cells in the nasal cavity were isolated and Alexa647-labeled OVA incorporated cells in CD11c^+^, CD326^+^, and CD8^+^CD11c^+^ cells were analyzed by flow cytometry (B-F). The gating strategy of each cell population in the nasal cavity is shown (B). Alexa647-labeled OVA incorporated cells in each cell fraction from non-treated mice (gray), OVA- (dashed line) and OVA-SF-10- (solid line) immunized mice are shown (C). “all” represents all Alexa647-labeled OVA incorporated cells and “high” represents high fluorescent intensity Alexa647-labeled OVA incorporated cells. After administration for 60 min, the mean fluorescent intensity (MFI) of Alexa647-labeled OVA in each population was analyzed by flow cytometry (D) (*n* = 5). Each bar represents the mean ± SEM of MFI of Alexa647-labeled OVA. The percentages of positive cells in all Alexa647-labeled OVA incorporated cells and percentages of high fluorescent intensity cells that incorporated large amounts of Alexa647-labeled OVA are shown in (E) and (F), respectively, (*n* = 5). *P < 0.05, **P < 0.01.

We reported previously that the natural bovine pulmonary surfactant medicine, Surfacten^®^ increases influenza HAv delivery efficiency to BMDCs and prolongs Ag clearance time in nasal cavity, resulting in enhancement of HAv-specific SIgA levels in nasal washes and HAv-specific IgG levels in serum of mice [12]. To determine the cell populations in the nasal mucosa responsible for the synthetic adjuvant SF-10-induced increase in Ag-delivery, we monitored incorporation of fluoresceinated OVA with or without SF-10 into nasal mucosa cells. After intranasal instillation of Alexa647-labeled OVA with or without SF-10 for 15 min, fluoresceinated OVA was found to be distributed extensively in the nasal cavity, and nasal mucosa layer was labelled with fluorescence more heavily by Alexa647-OVA-SF10 than Alexa647-OVA alone (Fig 1A). After instillation of fluoresceinated OVA or OVA-SF-10 for 60 min, mucosal cells were isolated, and Ag incorporation in CD11c^+^ cells (DCs), CD326^+^ cells (epithelial cells) and CD8^+^CD11c^+^ cells associated with induction of CTL was monitored by cellular fluorescent intensity (Fig 1B and 1C). The mean fluorescent intensities (MFI) were significantly higher (~3-fold) in OVA-SF-10-treated CD11c^+^ and CD326^+^ cells than those treated with OVA alone (*P* < 0.05). The MFI of OVA-SF-10-treated CD8^+^CD11c^+^ cells also tended to be higher (~4-fold) than that in the cells treated with OVA alone, although the difference was not significant (*P* = 0.077) (Fig 1D). The percentages of positive cells in all OVA incorporated cells were significantly higher in OVA-SF-10-treated CD11c^+^ and CD8^+^CD11c^+^ cells than those treated with OVA alone (Fig 1E). In addition, the percentage of high fluorescent intensity cells that incorporated large amounts of Alexa647-labeled OVA was also significantly higher in OVA-SF-10-treated CD11c^+^ and CD326^+^ cells than those treated with OVA alone (Fig 1F). Interestingly, SF-10 did not enhance Ag incorporation into B cells or T cells in nasal mucosa (data not shown).

### Ag-SSF induces activation of naïve CD8^+^ T cells mediated by cross-presentation in BMDC

**Fig 2:**
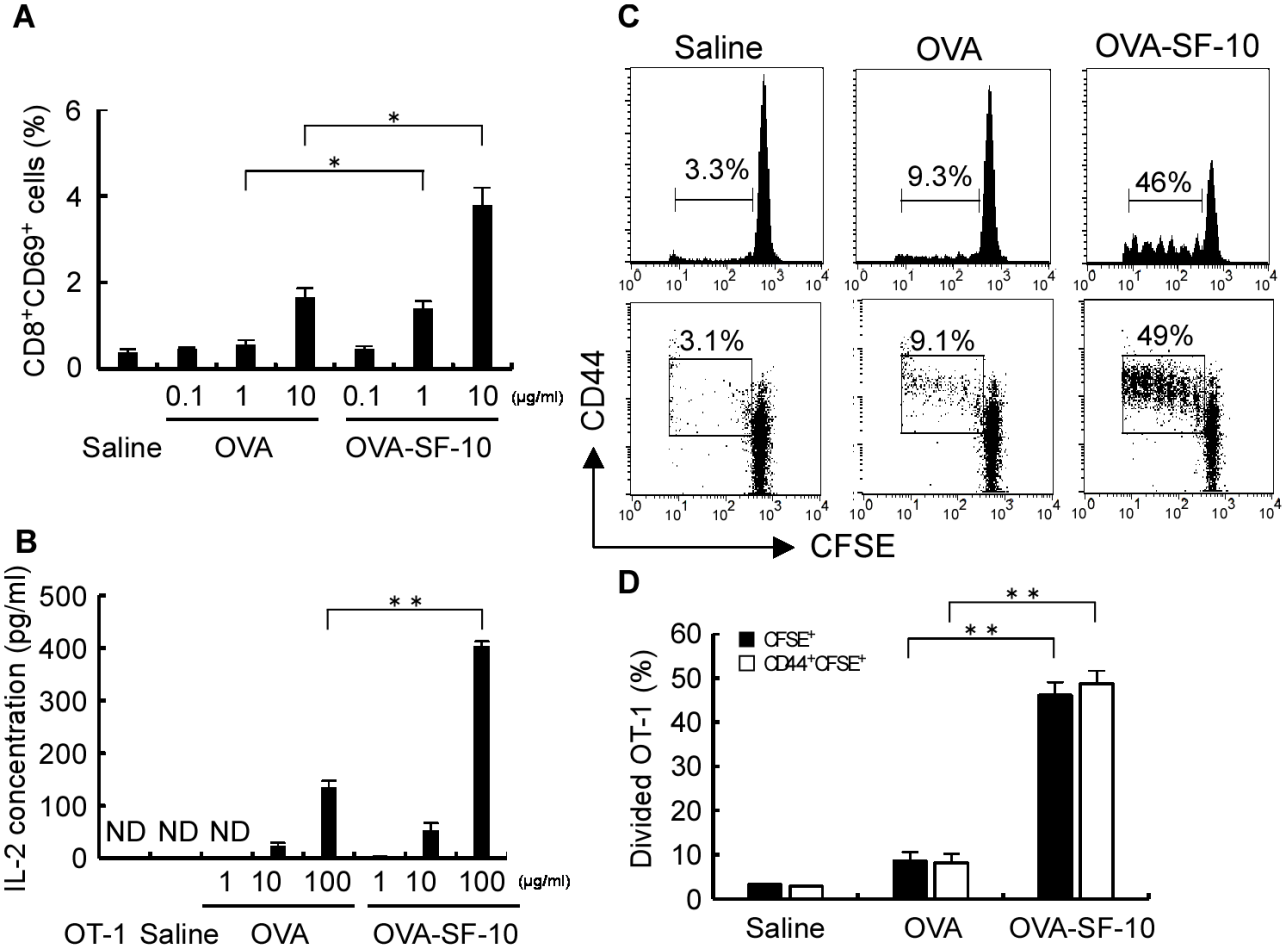
Activation of CD8^+^ T cells via Ag cross presentation both *in vitro* and *in vivo*. Splenocytes from OT-1 mice were stimulated with OVA or OVA-SSF for 1 h, and analyzed by flow cytometry the next day (A). Each bar represents the mean ± SEM of CD69^+^ cells on gated CD8^+^ cells (*n* = 3). To analyze primary CD8^+^ T cell activation by cross presentation *in vitro*, BMDCs from C57BL/6 were stimulated for 2 h with OVA or OVA-SSF (B). After washing the cells, the culture medium was replaced with fresh cRPMI and cultured for 24 h. The cells were then co-cultured with OT-1 CD8^+^ T cells for another 24 h. IL-2 concentrations in the culture medium after co-incubation were determined by ELISA. Each bar represents the mean ± SEM of IL-2 concentrations (*n* = 3). OT-1 cells were transferred intravenously into naïve C57BL/6 mice before nasal immunization and OVA, OVA-SF-10 (OVA: 10 μg) or saline. Lymphocytes from CLNs were collected at day 4 after immunization and analyzed by gating CFSE-positive cells (C, D). Each bar represents the mean ± SEM (*n* = 3). *P < 0.05, **P < 0.01. ND, not detected.

We next analyzed the effects of mucosal adjuvant SSF complexed with Ag on the activation of naïve CD8^+^ T cells from OT-1 mice. For *in vitro* experiments, we used SSF instead of SF-10 as an adjuvant, because Ag-SSF in the culture medium efficiently and freely contacts cultured cells without the viscosity improver CVP. Splenocytes of OT-1 mice were stimulated by OVA-SSF or OVA. OVA-SSF produced more efficient CD69 expression in CD8^+^ T cells, an early activation marker of CD8^+^ T cells, compared with OVA, and the effect was OVA-dose-dependent (Fig 2A).

To further investigate the role of DCs in OVA-SSF-induced CD8^+^ T cell activation, BMDCs from C57BL/6 mice were pulsed with OVA-SSF or OVA and then co-cultured with OT-1 CD8^+^ T cells for stimulation. Activation of OT-1 CD8^+^ T cells was assessed by quantification of IL-2 production in the conditioned medium. Although OVA treatment increased the secretion of IL-2 in a concentration-dependent manner, much higher levels of IL-2 were induced by OVA-SSF (*P* < 0.01) at a dose of 100 μg/ml OVA (Fig 2B). These results suggest that SSF activates naïve CD8^+^ T cells through up-regulation of Ag cross-presentation in DC.

### Ag-SF-10 promotes cross presentation and activates naïve CD8^+^ T cell proliferation *in vivo*

We examined the activation of OT-1 CD8^+^ T cells after an initial intranasal vaccination of mice with OVA-SF-10. CFSE-labelled OT-1 cells were adoptively transferred into naïve C57BL/6 mice, and then these mice were immunized with OVA-SF-10, OVA or saline intranasally 24 h after the treatment. Analysis of CFSE-labelled cells in the CLNs was performed 4 days after immunization (Fig 2C and 2D). OVA-SF-10 induced a significantly better proliferative response than OVA and saline, as monitored by the division rate of CFSE-labelled OT-1 CD8^+^ T cells. The phenotype of the proliferated OT-1 CD8^+^ T cells was determined by the expression of activation marker CD44. Dividing OT-1 CD8^+^ T cells expressed high levels of CD44 during proliferation and percentages of CD44^+^CFSE^+^ cells in the OVA-SF-10 group were about 5-fold higher than those in the OVA alone group (*P <* 0.01). Almost no proliferation was observed in saline group. These results indicate that intranasal administration of Ag-SF-10 promotes antigen presentation of APCs and induces proliferation of naïve CD8^+^ T cells *in vivo*.

### Ag-specific CTL induction in nasal mucosa

**Fig 3:**
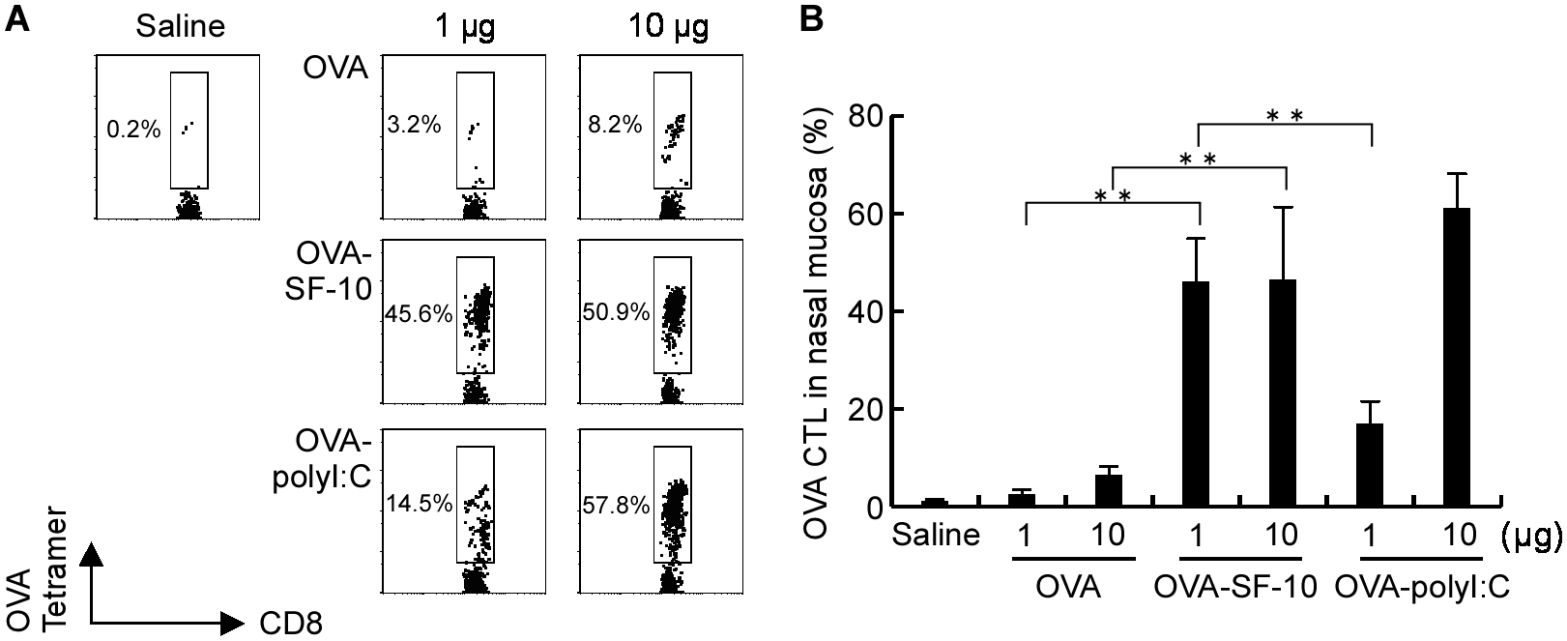
Detection of OVA-specific CTL in nasal mucosa. H-2K^b^ OVA tetramer^+^CD8^+^ T cells of nasal lymphocytes were measured after three times nasal immunization. C57BL/6 mice were immunized intranasally with OVA, OVA-polyI:C, or OVA-SF-10 (A). After the third immunization for 2 weeks, lymphocytes were prepared from nasal mucosa and antigen specific CTLs were determined by flow cytometry. Results (A) are representatives of four separate experiments. Cells were gated on CD8^+^ T lymphocytes. The bar graph of (B) shows mean ± SEM (*n* = 5) of the data in (A). Statistical significance was evaluated using one-way ANOVA with a Bonferroni posttest. **P < 0.01.

We investigated the induction of OVA-specific CTL, H-2K^b^ OVA tetramer^+^ cells in CD8^+^ T lymphocytes in the nasal mucosa after three courses of intranasal immunization with OVA-SF-10, OVA, or saline and OVA-polyI:C as a positive control [20], in C57BL/6 mice. The number of H-2K^b^ OVA tetramer^+^CD8^+^ T cells induced by OVA-SF-10 was significantly higher than those induced by OVA alone at two different doses of OVA (1 and 10 μg, Fig 3A and 3B). The number of OVA-specific CD8^+^ CTLs induced by OVA-polyI:C was also high and Ag-dose dependent, but the number of OVA-SF-10-induced CTLs reached a plateau at a lower dose of 1 μg OVA.

### Induction of CTL response and CTL-mediated cytolysis of target cells by HAv-SF-10

**Fig 4:**
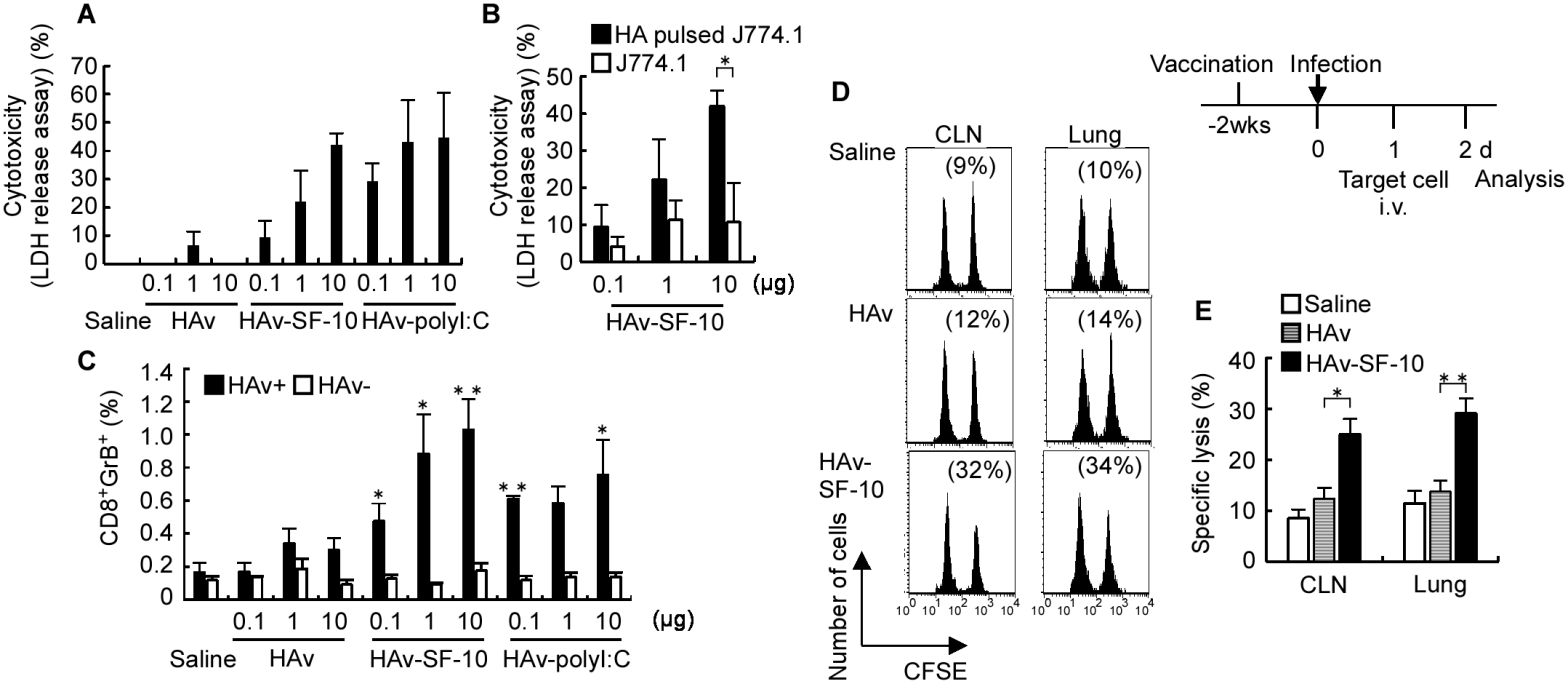
CTL-mediated cytolysis of target cells *in vitro* and *in vivo*. CTL activity against J774A.1 target cells pulsed with HA peptide was measured using LDH release assay (A and B). BALB/c mice were immunized three times intranasally with HAv, HAv-polyI:C or HAv-SF-10 at the indicated doses of HAv (from 0.1 to 10 μg), and splenocytes were isolated for restimulation with HAv and IL-2 for use as effector cells. After 72 h co-culture of the effector cells with the HA peptide-pulsed or non-pulsed J774A.1 target cells at effector: target ratio of 20:1 for 5 h, cytolysis of the target cells was evaluated by LDH release assay. Each bar represents the mean ± SEM (%) (*n* = 4). The splenocytes of BALB/c mice immunized 3 times intranasally with HAv, HAv-polyI:C or HAv-SF-10 were isolated, and incubated with or without 10 μg/ml HAv for stimulation. After 72 h, splenocytes were analyzed for GrB production by intracellular staining (C). GrB producing cells on gated CD8^+^ T cells were analyzed by flow cytometry. Each bar represents the mean ± SEM after incubation with HAv (+) or without HAv (-) in each immunization group (*n* = 4). *P*; versus the same dose of HAv. Two weeks (wks) after the third immunization, mice were infected with 100 × LD_50_ of PR/8, and then the target cells (5 × 10^6^) were equally mixed with HA peptide-unpulsed CFSE^low^ and pulsed CFSE^high^ splenocytes were injected intravenously (i.v.) at 24 h after infection. The cytolysis of peptide-pulsed CFSE splenocytes in CLNs and lungs was analyzed by flow cytometry and calculated according to the formula described in the *Materials and Methods* (D and E). Data are representative of separate experiments (D). Each bar represents the mean ± SEM of HAv-SF-10, HAv and saline in CLNs and lungs (E) (*n* = 6). *P < 0.05, **P < 0.01.

Induction of Ag-specific CTL is important for defense against infection [21]. To analyze the effects of nasal administration of HAv-SF-10 on influenza virus infection, we initially assessed the CTL-mediated cytolysis of target cells *in vitro*. J774A.1 cells pulsed with H-2K^d^-restricted HA peptide (533IYSTVASSL541) were used as target cells in the *in vitro* experiments. Splenocytes from BALB/c mice immunized intranasally three times with HAv-SF-10, HAv, saline or HAv-polyI:C were isolated, re-stimulated with HAv and IL-2 and used as effector cells. The effector cells in each group were co-cultured with the target cells at 20:1 ratio. Cytotoxicity was defined based on the amount of LDH released in the conditioned medium after incubation for 5 h (Fig 4A). Almost no CTL-mediated cytolysis was detected in the effector in mice treated with saline and HAv. In comparison, marked CTL-mediated cytolysis was detected in the effector in mice treated with HAv-SF-10 and HAv-polyI:C. HAv-dose-dependent cytolysis was clearly detected in the J774A.1 target cells pulsed with HA peptide but not without the treatment (Fig 4B).

CTL recognizes, binds and activates target cell death through exocytosis of cytotoxic granules, which contain perforin and granzyme (Gr). Gr cleaves cellular substrates, resulting in rapid and efficient cell death. To analyze the induction of CTL-mediated cytolysis by Ag-SF-10, we next evaluated the induction of GrB-expressing CD8^+^ T cells in splenocytes of BALB/c mice after immunization. Mice were immunized three times intranasally with HAv-SF-10, HAv, or saline and HAv-polyI:C and the splenocytes of each group were isolated 2 weeks after the third immunization, and then incubated with or without HAv at 10 μg/ml for 72 h. The percentage of CD8^+^GrB^+^ T cells among CD8^+^ T cells showed HAv-dose dependence (from 0.1 to 10 μg) and was significantly higher in mice immunized with HAv-SF-10 than HAv alone (Fig 4C). The percentage of CD8^+^GrB^+^ T cells was also significantly higher in mice immunized with an increasing doses of HAv with polyI:C at 10 μg compared with HAv alone, except for HAv dose of 1 μg. There was no significant difference in the percentage of CD8^+^GrB^+^ T cells between HAv and saline; the results being consistent with those shown in Fig 4A. In the absence of HAv for splenocyte stimulation, CD8^+^GrB^+^ T cells were not induced in all groups. The above results indicate that GrB production in CD8^+^ T cells is dependent on Ag stimulation and is enhanced by SF-10.

It has been reported that virus-specific CTL production is accelerated by memory T cells in secondary infection and that such enhancement is associated with reduced virus titers 2–3 days earlier comopared with naïve controls [19, 21]. We then analyzed CTL-mediated cytolysis *in vivo* in target infection organs, such as CLNs and lungs, of IAV infected mice, after intranasal immunization with HAv-SF-10. Two weeks after the third immunization of HAv-SF-10, HAv or saline, mice were infected with lethal dose at 100 × LD_50_ of IAV/PR/8/34 (PR/8) and then equal mix of HA peptide-pulsed CFSE^high^ and HA peptide-unpulsed CFSE^low^ splenocytes were transferred intravenously at 24 h post-infection. The percentage of cytolysis in CFSE-labelled splenocytes transfused in each group was analyzed by flow cytometry as described in the *Materials and Method*. The percentage of Ag-specific cytolysis was significantly higher in CLNs and lungs of mice immunized with HAv-SF-10 at the early phase of infection than those of mice immunized with HAv alone and saline (Fig 4D and 4E). These results indicate that nasal administration of HAv-SF-10 induces CTL-mediated cytolysis in the initial IAV infection respiratory organs.

### Effects of CD8^+^ and CD4^+^ T cell depletion in protective immunity against severe IAV infection in mice immunized with HAv-SF-10

**Fig 5:**
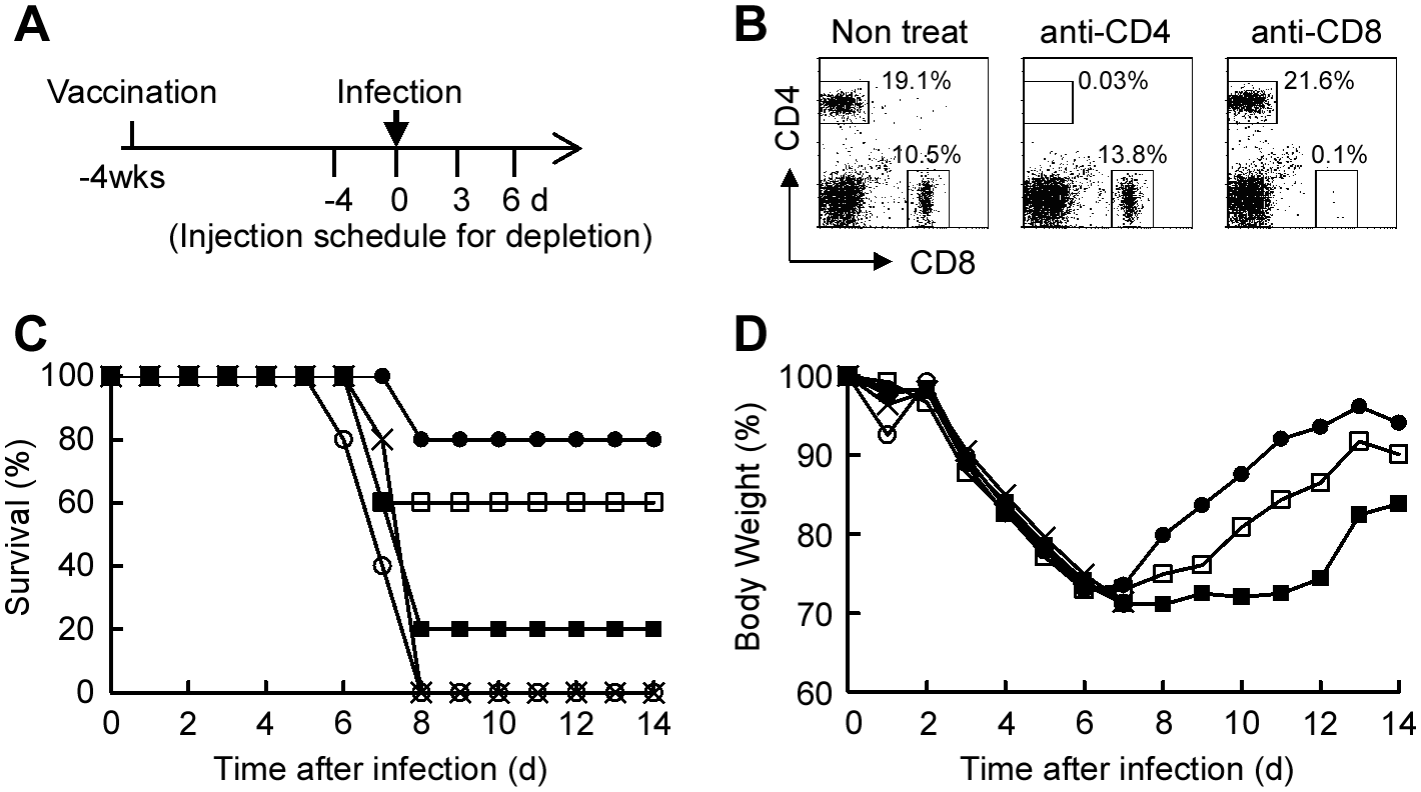
Effects of CD8^+^ and CD4^+^ T cell depletion on survival against severe infection with PR/8 in mice immunized intranasally with HAv-SF-10. Four weeks (wks) after the third intranasal immunization of BALB/c mice with HAv (**×**), HAv-SF-10 (●) and saline (**○**), each group (*n* = 5) was infected with a lethal dose of PR/8 at 20 × LD_50_. CD4^+^ (■) or CD8^+^ (**□**) T cell depletion in mice immunized with HAv-SF-10 was conducted by intraperitoneal injection (4 times) of 100 μg of anti-CD4 Ab (GK1.5) and anti-CD8α Ab (53–6.7), respectively, at the indicated time points (A). After the last depletion Ab injection for 4 days (d), CD4^+^ and CD8^+^ T cell counts in splenocytes were analyzed by flow cytometry (B). Survival rates (C) and changes in body weight (D) in each group of mice after PR8 challenge were monitored. The significance of changes in the survival rate was determined by the Kaplan-Meier method.

We reported previously that intranasal vaccination with HAv-SF-10 increases the survival rate and provides protection against body weight loss in mice infected with a lethal dose of PR/8 [13]. Recent findings demonstrated the important collaborative roles of both CD4^+^ and CD8^+^ T cell immunity in protection against IAV-mediated diseases [6]. In the present study, we analyzed the separate effects of CD4^+^ T cell depletion and CD8^+^ T cell depletion on protective immunity against IAV-induced severe pneumonia model in mice immunized with HAv-SF-10. BALB/c mice were immunized intranasally three times with HAv-SF-10, HAv or saline. Four weeks after the third immunization, mice were infected with a lethal dose of PR/8 (20 × LD_50_) in 20 μl saline, which is known to distribute deeply in the airways and causes severe pneumonia [22]. CD8^+^ and CD4^+^ T cells of the immunized mice were depleted by intraperitoneal injection (4 times) of anti-CD8α and anti-CD4, respectively, at the indicated time points around the infection time point and the effects of such depletion were confirmed after 4 days of the last injection (Fig 5A and 5B). Although all mice treated with saline and HAv alone died within 8 days after infection, 80% of mice immunized with HAv-SF-10 survived (*P* = 0.010, vs saline; *P* = 0.015, vs HAv), and these mice showed rapid recovery of body weight loss, which was observed from day 7 after infection. On the other hand, mice immunized with HAv-SF-10 and then depleted of CD8^+^ T cells in the severe pneumonia model showed a mild decrease in the survival rate from 80 to 60% (*P* = 0.364, vs without depletion) (Fig 5C), and also showed mild delay in recovery of body weight loss (Fig 5D). In contrast, mice immunized with HAv-SF-10 and then depleted of CD4^+^ T cells showed a clear decrease in survival rate from 80 to 20% (*P* = 0.052, vs without depletion) (Fig 5C), and also a clear delay of recovery in body weight loss (Fig 5D).

## Discussion

The present study describes several new findings on cell-mediated immunity induced by mucosal adjuvant SF-10. These findings include i) Nasal administration of Ag-SF-10 induced abundant Ag-specific CTL in nasal mucosa and spleen. ii) SF-10 stimulated Ag-delivery to APCs and epithelial cells of the nasal mucosa, activated CD8^+^ T cells through up-regulation of cross-presentation, and induced CTL-mediated cytolysis in the lungs and CLNs in the early phase of infection. iii) CD8^+^ T cell-mediated cytolysis, which plays a role in limiting viral spread, was significantly enhanced by SF-10 in the early phase of IAV infection and HAv-SF-10-induced CD8^+^ T cells were partially while CD4^+^ T cells were predominantly involved in the survival of mice in the advanced stage of severe pneumonia.

In the present study, we found that intranasal inoculation of Ag-SF-10 resulted in high level of CTL (Ag-specific Tetramer^+^CD8^+^ T cells) induction in the nasal mucosa (Fig 3). The CTL induced by intranasal administration of HAv-SF-10 was responsible for cytolysis in the target cells pulsed with HA peptide in the spleen, and was also responsible in the lungs and CLNs at the early phase of IAV infection (Fig 4). The respiratory mucosa is the site of invasion of microbial pathogens, hence, the induction of high levels immune responses in the nasal and lung mucosa is the main defense strategy for airway infection. Not only humoral immune responses, but also systemic and local cell-mediated immune responses by CTL, are important for protection and prevention.

It has been reported that a relatively high dose of Ag is required to induce CD8^+^ T cell response [23, 24]. Administration of Ag alone in the nasal cavity is usually associated with poor efficiency of Ag uptake across the mucosa due to mucociliary clearance, resulting in weak cell-mediated immune response [7, 8, 25]. To enhance Ag delivery, many types of liposomes have been proposed as delivery carriers [26], and these vehicles are reported to induce cell-mediated immunity both *in vitro* and in the spleen [27], but little report on local tissue-resident CD8^+^ T cells. We reported previously that pulmonary surfactant has liposome-like characteristics, and when used as a delivery vehicle, it delivers Ag to BMDC [12]. Both natural pulmonary surfactant and its synthetic compound SF-10 prolong the duration of Ag in nasal cavity and delivers the Ag efficiently to the epithelial cells as well as deeply-located APCs in the nasal mucosa [12] (Fig 1). In comparison with administration of Ag alone, we found that administration of Ag-SF-10 delivered significantly higher doses of Ag to CD11c^+^ DCs, and importantly to the CD8^+^ CD11c^+^ DCs subset in nasal mucosa, which is known to dedicate cross presentation for activation of CD8^+^ T cells [28].

SF-10 and SSF have the advantages of efficient Ag delivery to APCs. These adjuvants have a cationic transmembrane lipopeptide K6L16, which mimics the SP-C, and its hydrophobic tail is buried in the lipid vesicle. The cationic N-terminal segment provides positive charge to the vesicle surface, which may play an important role in the interaction between Ag-SF-10/SSF and the negatively charged phosphate group of the plasma membrane of APCs and epithelial cells present in the nasal mucosa, resulting in efficient interaction between the two and Ag delivery effects. Ag delivery into APCs by SF-10 and SSF may also play a role in MHC class I presentation by an efficient access to the Ag processing pathways in the cytosol. In addition, recent studies described a new pathway named “cross-dressing” thought, which might be involved in cell-mediated immunity [29, 30]. In this pathway, Ag peptide-MHC class I complexes are transferred to DCs from other nearby cells, including epithelial cells. Although the role of “cross-dressing” activity of epithelial cells in the cross-presentation in DCs has not been clarified, an efficacious antigen trapping in mucosal epithelial cells in the nasal cavity (Fig 1) may enhance Ag-SF-10-induced CD8^+^ T cell activation.

The tissue location of persisting resident CD8^+^ memory T cells has robust protective capacities to site-specific infection [31], such as lung resident CD8^+^ memory T cells for enhanced protective immunity against influenza infection [32]. In addition, it has been reported that intranasal administration of influenza vaccine induces lung resident CD8^+^ memory T cells and provides heterosubtypic protection to lower respiratory tract influenza infection in mice [33, 34]. However, these results were from the data in a lower respiratory tract vaccination model in which mice were administrated intranasally 20 μl or more of vaccine that reach the lower respiratory tract, but not the results of a nasal cavity vaccination model. In this study, we used nasal cavity vaccination as a model of clinical vaccination, in which mice were administrated intranasally 6 μl of HAv-SF-10 (3 μl in each nostril). The results indicated that intranasal administration of Ag-SF-10 stimulated APCs and CD8^+^ T cells in nasal passage (Figs 1 and 3) and induced CTL (Fig 4) in the lungs. These results suggest that SF-10 can induce cell-mediated immunity in both upper and lower respiratory tracts by vaccination in nasal passage.

CD8^+^ and CD4^+^ T cells have distinct but important collaborative roles in the clearance of IAV infection [35]. CD4^+^ helper T cells promote effective immunity by providing the secondary signals for antibody responses and proinflammatory cytokine production upon infection. On the other hand, CD8^+^ T cells locate and kill virus-infected cells, thus limiting viral spread at the initial IAV infection sites. As expected, CD8^+^ T cell-mediated cytolysis was markedly enhanced by SF-10 in the early phase of IAV infection (Fig 4), and effects of CD8^+^ and CD4^+^ depletion in mice immunized with HAv-SF-10 showed that CD4^+^ T cells predominantly while CD8^+^ T cells partially contributed to mice survival in the advanced stage of severe pneumonia (Fig 5).

In conclusion, we have demonstrated in the present study that SF-10 plays an important role as a mucosal adjuvant in the induction of cell-mediated immunity in addition to the reported humoral immunity and is a useful mucosal adjuvant for nasal vaccination.
